# Chinese Herbal Medicine Combined With EGFR-TKI in EGFR Mutation-Positive Advanced Pulmonary Adenocarcinoma (CATLA): A Multicenter, Randomized, Double-Blind, Placebo-Controlled Trial

**DOI:** 10.3389/fphar.2019.00732

**Published:** 2019-07-02

**Authors:** Lijing Jiao, Jianfang Xu, Jianli Sun, Zhiwei Chen, Yabin Gong, Ling Bi, Yan Lu, Jialin Yao, Weirong Zhu, Aihua Hou, Gaohua Feng, Yingjie Jia, Weisheng Shen, Yongjian Li, Ziwen Zhang, Peiqi Chen, Ling Xu

**Affiliations:** ^1^Institute of Clinical Immunology, Yueyang Hospital of Integrated Traditional Chinese and Western Medicine, Shanghai University of Traditional Chinese Medicine, Shanghai, China; ^2^Department of Oncology, Yueyang Hospital of Integrated Traditional Chinese and Western Medicine, Shanghai University of Traditional Chinese Medicine, Shanghai, China; ^3^Department of Oncology, Shanghai Pulmonary Hospital, Tongji University, Shanghai, China; ^4^Department of Oncology, Longhua Hospital, Shanghai University of Traditional Chinese Medicine, Shanghai, China; ^5^Lung Tumor Clinical Medical Center, Shanghai Chest Hospital, Shanghai Jiaotong University, Shanghai, China; ^6^Department of TCM, Ruijin Hospital, Shanghai Jiaotong University, Shanghai, China; ^7^Department of Oncology, Yantai Hospital of Traditional Chinese Medicine, Yantai, China; ^8^Department of Oncology, Zhangjiagang Hospital of Traditional Chinese Medicine, Zhangjiagang, China; ^9^Department of Oncology, First Teaching Hospital of Tianjin University of Traditional Chinese Medicine, Tianjin, China; ^10^Department of Oncology, Jiangsu Jianyin People’s Hospital, Jiangyin, China; ^11^Department of Oncology, Suzhou Hospital of Traditional Chinese Medicine, Suzhou, China; ^12^Department of Oncology, Changshu No.2 People’s Hospital, Changshu, China; ^13^Tumor Institute of Traditional Chinese Medicine, Chinese Medicine Research Institute, Shanghai University of Traditional Chinese Medicine, Shanghai, China

**Keywords:** pulmonary adenocarcinoma, EGFR activating mutations, EGFR-TKI, Chinese herbal medicine, drug resistance

## Abstract

**Background:** To determine the clinical activity and safety of Chinese herbal medicine (CHM) combined with epidermal growth factor receptor tyrosine kinase inhibitors (EGFR-TKI) in patients with advanced pulmonary adenocarcinoma (ADC) and the ability of CHM combined with EGFR-TKI to activate EGFR mutations.

**Methods:** Three hundred and fifty-four patients were randomly assigned to EGFR-TKI (erlotinib 150 mg/d, gefitinib 250 mg/d, or icotinib 125 mg tid/d) plus CHM (TKI+CHM, *N* = 185) or EGFR-TKI plus placebo (TKI+placebo, *N* = 169). Progression-free survival (PFS) was the primary end point; the secondary end points were overall survival (OS), objective response rate (ORR), disease control rate (DCR), quality of life [Functional Assessment of Cancer Therapy-Lung (FACT-L) and Lung Cancer Symptom Scale (LCSS)], and safety.

**Results:** The median PFS was significantly longer for the TKI+CHM group (13.50 months; 95% CI, 11.20–16.46 months) than with the EGFR-TKI group (10.94 months; 95% CI, 8.97–12.45 months; hazard ratio, 0.68; 95% CI, 0.51–0.90; *P* = 0.0064). The subgroup analyses favored TKI+CHM as a first-line treatment (15.97 *vs.* 10.97 months, *P* = 0.0447) rather than as a second-line treatment (11.43 *vs.* 9.23 months, *P* = 0.0530). Patients with exon 19 deletion had a significantly longer PFS than with 21 L858R. The addition of CHM to TKI significantly improved the ORR (64.32% *vs.* 52.66%, *P* = 0.026) and QoL. Drug-related grade 1–2 adverse events were less common with TKI+CHM.

**Conclusions:** TKI+CHM improved PFS when compared with TKI alone in patients with EGFR mutation-positive advanced non-small-cell lung cancer (NSCLC).

Clinical Trial Registration: www.ClinicalTrials.gov, identifier NCT01745302.

## Introduction

For advanced non-small-cell lung cancer (NSCLC) harboring epidermal growth factor receptor (EGFR) activating mutations (mainly exon 19 Del and exon 21L858R), EGFR tyrosine kinase inhibitors (EGFR-TKIs) have been shown to result in superior outcomes when compared with platinum-based chemotherapy (Mok et al., 2009; Mitsudomi et al., 2010; Zhou et al., 2011; Rosell et al., 2012; Shi et al., 2017). Despite initial positive responses to EGFR-TKIs, most patients will have acquired resistance to the EGFR-TKIs, and the disease will progress within 9–14 months (Mok et al., 2009; Rosell et al., 2009; Mitsudomi et al., 2010; Zhou et al., 2011; Rosell et al., 2012; Shi et al., 2017). Although osimertinib has been shown to be effective for acquired resistance because of exon20 T790M, resistance to osimertinib tends to develop within a year (Mok et al., 2017) and is hard to address (Thress et al., 2015). Thus, optimizing the effect of EGFR-TKI is essential for the long-term survival of NSCLC patients. Efforts have been made to delay the acquired resistance to EGFR-TKI, such as adding chemotherapy to EGFR-TKI treatments (Cheng et al., 2016) or using osimertinib as the initial treatment for advanced NSCLC with sensitizing EGFR mutations (Ramalingam et al., 2018). However, these “overdraft” strategies might not benefit the patient’s overall survival (OS) and may be related with increased toxicity (Lee et al., 2017).

The yin-yang theory provides a macroscopic view of biological phenomena. Various cancer-associated genes and proteins have been reported to regulate various types of cancers in a yin-yang manner (Wei, 2018). Traditional Chinese medicine (TCM) views diseases as an imbalance between yin and yang, and the Chinese theory of yin-yang has been used in treatments, specifically making Chinese herbal medicine (CHM) an alternative therapy for NSCLC (Li et al., 2013; Han et al., 2016; Jiao et al., 2017).

The efficacy of CHM in combination with EGFR-TKI in delaying acquired resistance and prolonging PFS and OS has been demonstrated in several clinical trials (Yang et al., 2014; Hung et al., 2017; Yang et al., 2017). However, these trials have either been retrospective or have had small samples, and the influence of CHM on the T790M mutation has never been studied.

The current CATLA study was to determine whether the addition of CHM to EGFR-TKI (TKI+CHM) prolongs PFS compared with EGFR+placebo (TKI) in advanced pulmonary adenocarcinoma (ADC) patients who have an activating EGFR mutation.

## Patients and Methods

### Patients

In the current study, the inclusion criteria are as follows: 1) pathologically or cytologically confirmed with stage IIIa–IV with ADC; 2) patients with the mutated EGFR will be subjected to the first-line target therapy; patients who have received at least one cycle platinum-containing chemotherapy regimens with disease progression/recurrence or intolerant/refuse and who have proceeded to chemotherapy will explore the second-line target therapy; 3) Eastern Cooperative Oncology Group (ECOG) performance status (PS) scores of 0, 1, or 2; 4) age ≥ 18 years; 5) estimated life expectancy of at least 12 weeks; 6) no major organ dysfunction.

Patients were excluded if they had already received the targeted treatment (e.g., EGFR-TKI) or other anticancer treatments, such as immunotherapy or biologic therapy. Indeed, patients with symptomatic brain metastases should receive radiotherapy before enrollment, so palliative irradiation of bone lesions was allowed.

### Study Design and Treatment

This multicenter, randomized, double-blind, placebo-controlled study compared first-generation EGFR-TKIs (gefitinib, erlotinib, or icotinib)+CHM versus EGFR-TKIs+placebo as the first- or second-line therapy in Chinese patients with advanced ADC and who had EGFR activating mutations. There were 15 sites in China at which the patients were recruited. All of the patients were given informed consent prior to conducting the study. The study procedures and informed consent form were approved by the Institutional Review Board of Longhua Hospital in Shanghai [Institutional Review Board (IRB) no. 2012LCSY018], and until May 28, 2018, the study will still be reviewed by IRB.

### Random Assignment

Patients were randomly assigned to receive either EGFR-TKI+CHM (TKI+CHM) or EGFR-TKI (TKI) at a ratio of 1:1. The minimization method was implemented *via* central randomization on the Internet by a clinical research organization (CRO) (Shanghai Clinical Research Center, Shanghai, China); the patients were stratified by sex (male *vs.* female), age (<65 *vs.* ≥65), ECOG PS (0 *vs.* 1 *vs.* 2), staging (IIIa *vs.* IIIb *vs.* IV), smoking status (yes *vs.* no), first or second line, EGFR mutation status (19 Del *vs.* 21L858R *vs.* other rare mutations), TKIs (gefitinib *vs.* erlotinib *vs.* icotinib), and by which center the patient was recruited from. During the study, clinicians and subjects were blinded to the type of treatment being given.

### Treatment Protocol

Patients assigned to TKI+CHM received oral EGFR-TKI [erlotinib (Roche, Switzerland) 150 mg, gefitinib (AstraZeneca, UK) 250 mg, or icotinib (Beta, China) 125 mg per dose, three times per day; the drug was chosen by the patients] plus oral CHM.

### TCM Syndrome Differentiation and CHM Preparation

CHM was chosen from three prescriptions based on TCM syndrome differentiation: a qi-benefiting recipe, a yin-nourishing recipe, and a qi-yin benefiting recipe. One experienced TCM physician was assigned to differentiation syndromes and collected the baseline information. TCM diagnostic data and the prescribed medication for 1 month were given at the first visit. The bioactive components were extracted from water. Some quality control markers were selected by referring to the Chinese Pharmacopoeia (2015 edition), and the quantitative analysis was performed by high-performance liquid chromatography coupled with ultraviolet detection (HPLC-UV). The results were as follows: calycosin-7-O-beta-D-glucoside 0.012%, icariin 0.075%, psorale isopsoralen 0.067% in formula I, rosmarinic acid 0.036%, and apigenin 0.0069% in formula IV, which provided the quality control of the formula. The three formulas were applied according to the following regimens **(** **Supplementary Figure 1** **,** **Table 1** **)** (Wang et al., 2018):

1. *Formula I* (including tonifying qi and warming yang granules) **(** **Supplementary 1** **)**.
2. *Formula II* (nourishing yin granules) **(** **Supplementary 2** **)**.
3. *Formula III* (yin-nourishing and qi-tonifying granules).
  Formula III was a combination of formula I and formula II.
4. *Formula IV* (detoxifying and resolving masses granules) **(** **Supplementary 3** **)**.

**Table 1:** Traditional Chinese medicine (TCM) syndrome differentiation.*

TCM syndrome differentiation	Main symptoms	Tongue diagnosis	CHM regimens
1. *Qi syndrome deficiency*	Cough, large amount of sputum, loss of appetite, fatigue and weakness, pale and bulgy tongue. Secondary symptoms: spontaneous sweat, unshaped stool, thin superficial, and smooth pulse.	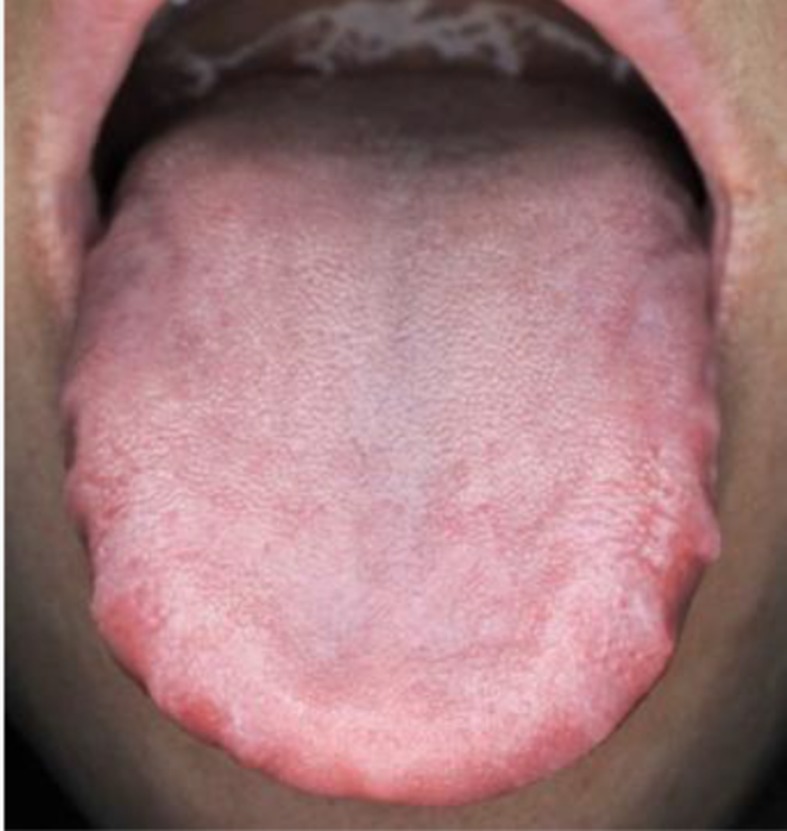	*Formula I + formula IV*
2. *Yin syndrome deficiency*	Cough, small amount of sputum, dried mouth, red tongue. Secondary symptoms: night sweats, insomnia, low fever, thready pulse, rapid pulse.	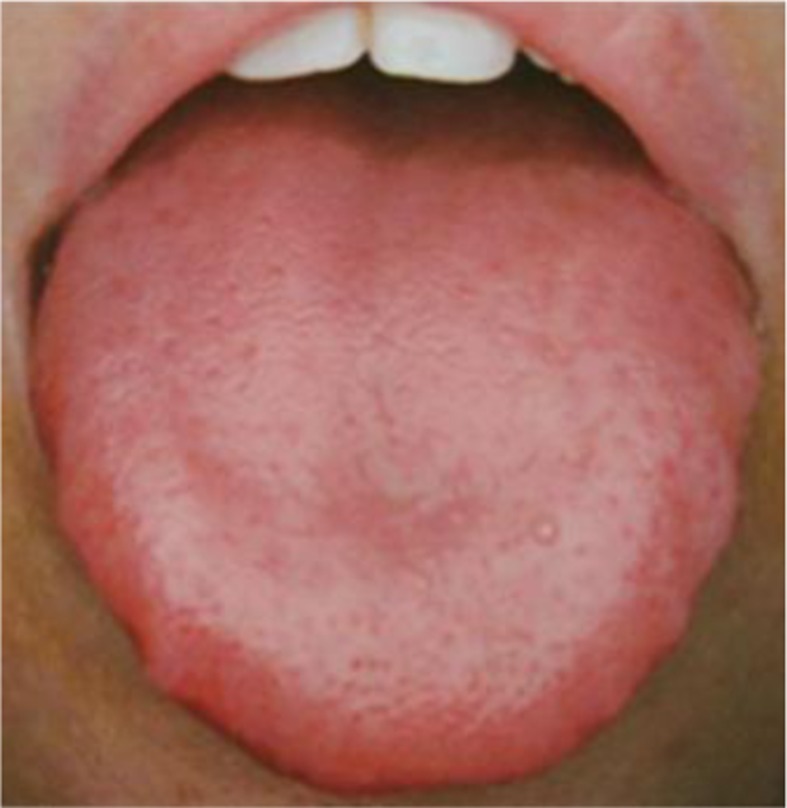	*Formula II + formula IV*
3. *Qi and yin syndrome deficiency*	Cough, small amount of sputum, fatigue and weakness, dried mouth without polydipsia. Secondary symptoms: spontaneous sweat, night sweets, reddish tongue or tongue with teeth imprints, thready and weak pulse.	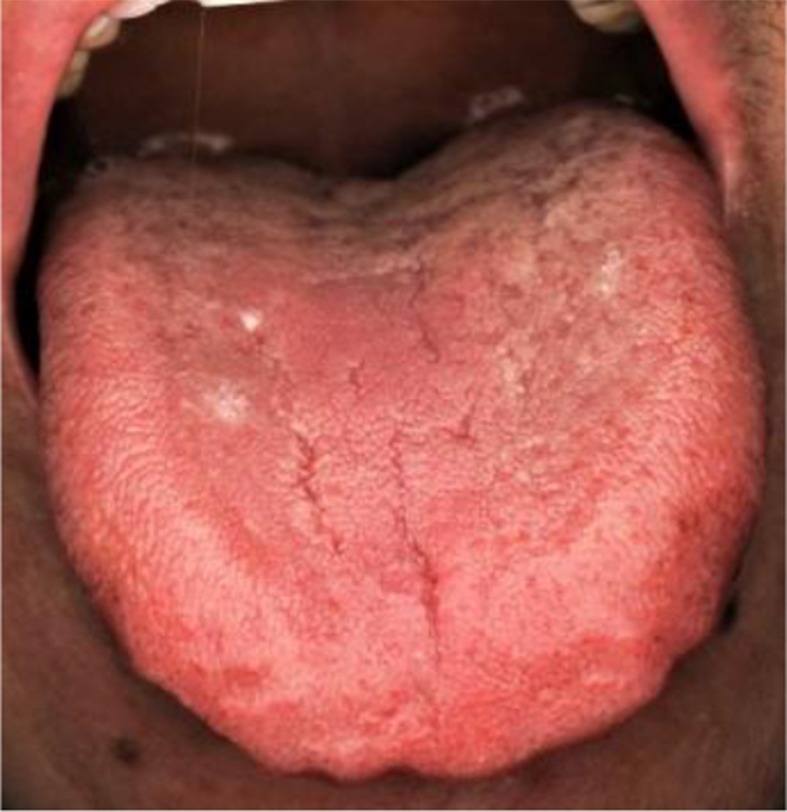	*Formula III + formula IV*

*Based on the Chinese Medicine New Medicine Clinical Practice Guideline (trial implementation) (published by China Medical Science Press in 2002) and Shanghai Chinese Medicine Routine Practice (written by the Shanghai Municipal Commission of Health and Family Planning).

We used the same method to prepare CHM and the placebo granules as previously reported (Wang et al., 2018). CHM granules (batch numbers 1208304, 1208303, and 1207357) were manufactured by Tianjiang Pharmaceutical Co., Ltd. (Jiangyin, Jiangsu Province, China) according to the good manufacturing practice (GMP) requirements. Three kinds of oral placebos were matched by weight, color, smell, taste, and packaging according to the three CHM formulas. The placebo was produced with flavorings and food by the same manufacturer but without the medical ingredients. The patients took the Chinese medicine applications on the same day as EGFR-TKI. The CHM granules were dissolved into 150 ml of warm water to drink twice per day after a meal until the end of the EGFR-TKI treatment. Clinical research pharmacists took part in and supervised the procedures.

EGFR-TKI and CHM treatment lasted until disease progression, unacceptable toxicity, or any other study discontinuation criteria were met. According to previous studies on drug-related toxicities (Melosky and Hirsh, 2014; Califano et al., 2015), dose adjustments or delays were implemented. For EGFR-TKI-treated patients with serious diarrhea, rashes, or any other EGFR-TKI-related adverse event (AE), the dosage could be stopped for up to 14 days, and appropriate symptomatic treatment could be provided. EGFR-TKI was stopped once interstitial lung disease occurred. For CHM-treated patients with abnormal liver function, poor appetite, nausea, diarrhea, and other gastrointestinal AEs, CHM could be delayed up to 14 days.

### Outcome Measures

The primary endpoint was PFS, which was measured with the date of the videography from a random assignment to the date of objective progression or death by the researcher. The secondary endpoints included a comparison of OS, ORR, DCR, quality of life (QoL), and safety.

Computed tomography or magnetic resonance imaging were used to assess tumor at baseline and every 8 weeks until disease progression. Patients who received more than 80% of the expected dose of EGFR-TKI and CHM were considered adherent. We recorded treatment-emergent AEs (TEAEs) per the Medical Dictionary for Regulatory Activities (version 15.0) and graded them using the National Cancer Institute’s common terminology criteria for adverse events (version 3.0).

QoL was collected using the Functional Assessment of Cancer Therapy–Lung (FACT-L) questionnaire (Cella et al., 2002; Thongprasert et al., 2011) and the Lung Cancer Symptom Scale (LCSS) (Hollen et al., 1999).

### Statistical Analysis

All patients were randomly assigned to treatment groups and had received at least one dose of CHM to be counted in the efficacy analyses. The safety population consisted of all patients (418 cases), including 64 EGFR-wide-type patients who had received at least one dose of the study drug.

The primary PFS end point was used to estimate the sample size. Approximately 95 PFS events were expected if 274 patients were enrolled (1:1), here with an inspection level of α = 0.05 and power 1-β = 0.80, hazard ratio (HR) = 0.56 for TKI+CHM versus TKI alone, corresponding to a 7-month improvement in PFS based on previous trials using CHM or TKI alone in the first-line therapy. The PFS of the second-line EGFR-TKI targeted therapy was 7 months (Kim et al., 2008), with an inspection level of α = 0.05 and power 1-β = 0.80, HR = 0.50; here, CHM plus EGFR-TKI may increase PFS to 12 months in the second-line therapy of advanced lung adenocarcinoma with EGFR mutation. There were 129 cases after calculation (64 cases in each group). It took 18 months to recruit the patients, and the research lasted for 36 months. Because of a 10% expulsion rate, there were a total of 443 cases in the study.

PFS is illustrated by the Kaplan–Meier survival curve and log-rank test. For the PFS and tumor response, an adjusted Cox regression model was used to estimate the adjusted HRs for differences between the treatment arms with the selected prognostic factors, including the EGFR mutation type, age, sex, EGFR-TKI drugs, treatment line, smoking status, and ECOG PS. Fisher’s exact test was used to compare tumor response rates and the incidence of TEAEs between the arms. *P* < 0.05 was considered statistically significant. Analyses were performed using SAS version 9.2 (SAS Institute, Cary, NC). Shanghai Clinical Research Center (SCRC) experts were invited to manage the statistical analysis.

## Results

### Patient Disposition

**Figure 1:**
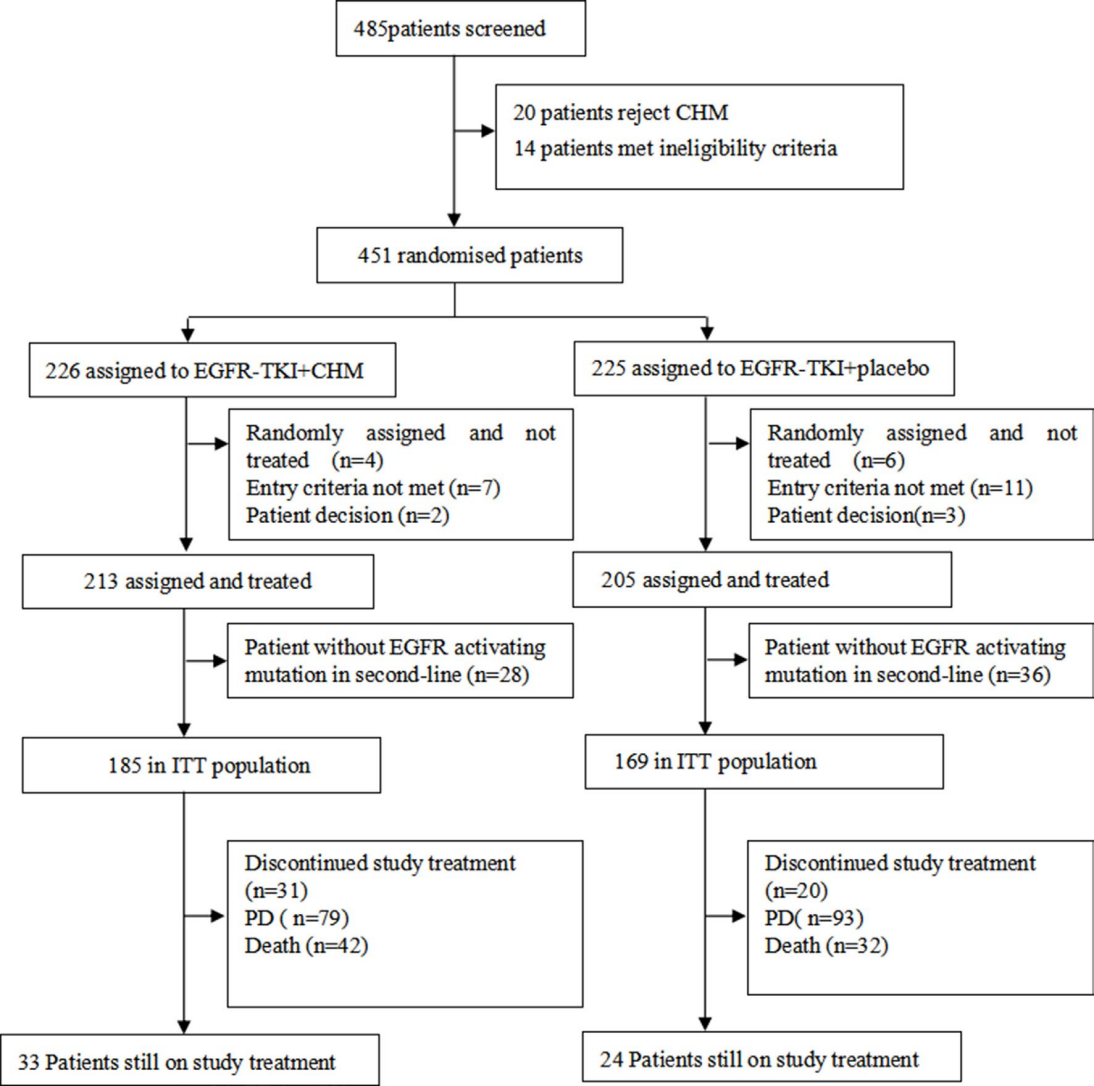
CONSORT diagram: trial profile at the cut-off date for analysis (January 5, 2018) PD, progressive disease.

In the current study, 451 patients were enrolled and randomized, and 418 patients were included in full analysis set, including 64 patients with an unknown type of EGFR in the second-line therapy; these 64 patients were excluded from the final analysis (December 28, 2012 to August 22, 2016). For the present study, 354 patients with EGFR activating mutations received at least one dose of the treatments used for the study and were assigned to either TKI+CHM (*N* = 185) or TKI (*N* = 169; **Figure 1**). Demographic and baseline clinical characteristics were matched, save for the clinic stage between the arms **(** **Table 2** **)**. In the TKI+CHM arm, the mean number for completed treatment was 12.62 (range, 0–52 months) and 10.75 (range, 0–38 months) in the TKI arm. The mean relative dose-intensity in the TKI+CHM arm was 98.92% and 89.18% for EGFR-TKI and CHM, respectively, and 98.22% (EGFR-TKI) and 86.98% (placebo) in the TKI arm. There was no difference in the proportion of patients adhering to TKI in both arms, 99.45% (184 of 185 patients) in the TKI+CHM arm and 98.82% (167 of 169 patients) in the TKI arm.

**Table 2:** Patient demographic and baseline disease characteristics [intent-to-treat (ITT) population].

Characteristic	EGFR-TKI+CHM No. of patients (%)	EGFR-TKI+placebo No. of patients (%)	Total
	(*N* = 185)	(*N* = 169)	354
Age, years			
Mean (SD)	61.3 ± 9.3	60.7 ± 10.8	61.0 ± 10.0
Age group			
<65 years	107 (57.80)	105 (62.10)	212 (59.90)
≥65 years	78 (42.20)	64 (37.90)	142 (40.10)
Sex			
Male	70 (37.84)	60 (35.50)	130 (36.72)
Female	115 (62.16)	109 (64.50)	224 (63.28)
Disease stage			
IIIa	7 (3.78)	4 (2.37)	11 (3.11)
IIIb	15 (8.11)	4 (2.37)	19 (5.37)
IV	163 (88.11)	161 (95.27)	324 (91.53)
Histology			
Adenocarcinoma	185 (100)	169 (100)	354 (100)
Smoking status§			
Never-smokers	153 (82.70)	132 (78.10)	285 (80.50)
Present smokers and light former smokers	32 (17.30)	37 (21.90)	69 (19.50)
EGFR-TKI therapy			
First line	125 (67.57)	124 (73.37)	249 (70.34)
Second line	60 (32.43)	45 (26.63)	105 (29.66)
EGFR-TKI drugs			
Gefitinib	81 (43.78)	69 (40.83)	150 (42.37)
Erlotinib	6 (3.24)	6 (3.55)	12 (3.39)
Icotinib	98 (52.97)	94 (55.62)	192 (54.24)
ECOG PS score			
0	11 (5.95)	7 (4.14)	18 (5.08)
1	167 (90.27)	153 (90.53)	320 (90.40)
2	7 (3.78)	9 (5.33)	16 (4.52)
CHM syndrome			
Qi deficiency	66 (35.68)	70 (41.42)	136 (38.42)
Yin deficiency	29 (15.68)	26 (15.38)	55 (15.54)
Qi and yin deficiency	90 (48.65)	73 (43.20)	163 (46.05)
EGFR mutation type			
Exon 18 G719X point mutation	1 (0.54)*	3 (1.78)	4 (1.13)
Exon 19 deletion	95 (51.35)	99 (58.58)	194 (54.80)
Exon 20 insertion	1 (0.54)**	0 (0.00)	1 (0.28)
Exon 21 L858R point mutation	88 (47.57)	67 (39.64)	155 (43.79)

*18G719X+19del; **19del+20ins.

§never-smokers (100 cigarettes in their lifetime); light former smokers (stopped smoking 15 years previously and smoked 10 pack-years); present smoker defined as someone who had smoked more than 100 cigarettes in their lifetime or who was either currently smoking or had stopped smoking less than 15 years or more ago.

### Clinical Activity

**Figure 2:**
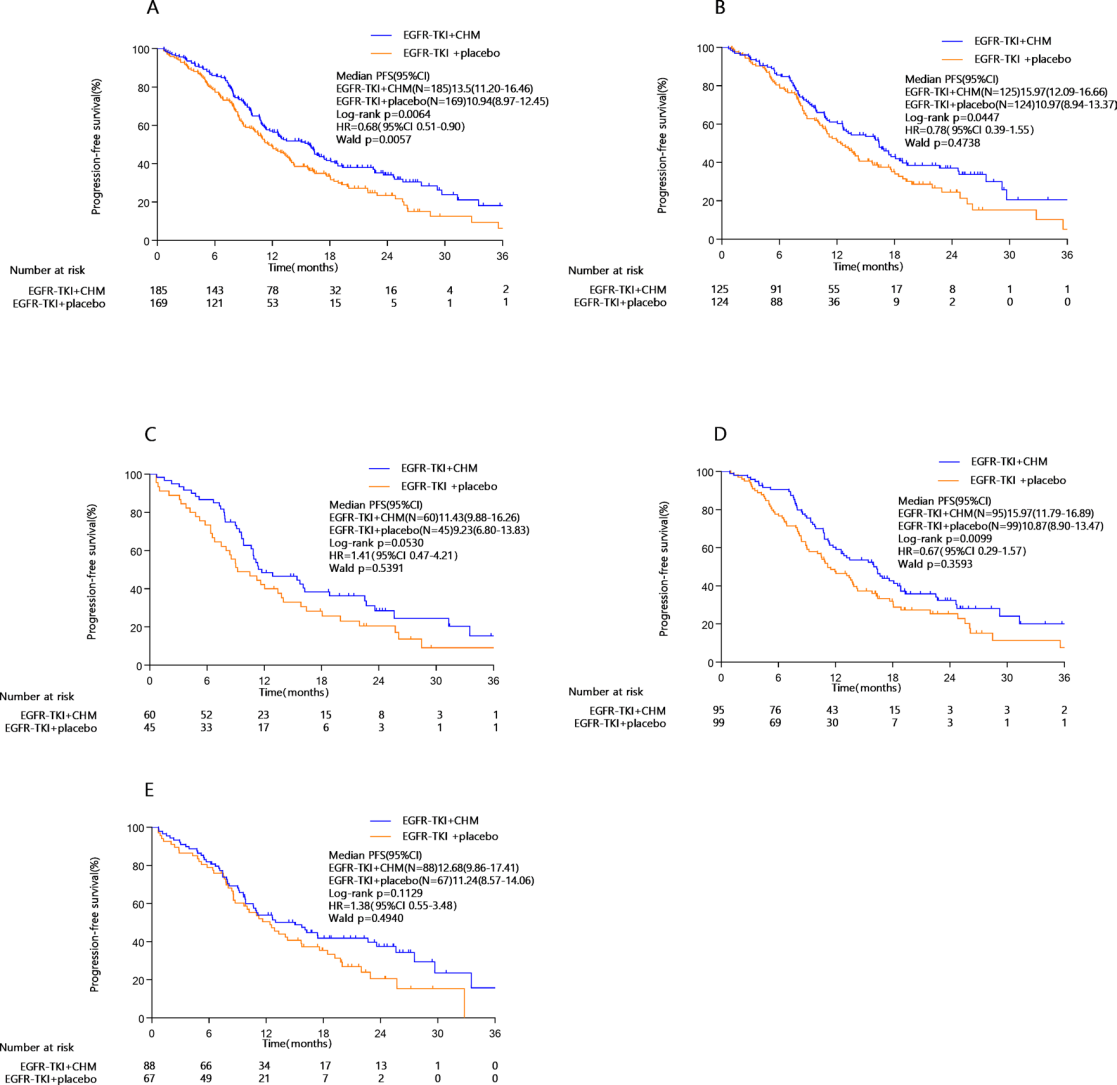
PFS in the **(A)** intent-to-treat population, **(B)** first-line EGFR-TKI subgroup, **(C)** second-line EGFR-TKI subgroup, **(D)** exon 19 deletion subgroup, **(E)** 21 L858R point mutation subgroup; PFS, progression-free survival; EGFR-TKI, epidermal growth factor receptor tyrosine kinase inhibitors.

At the end of the study period (January 2018), 207 patients (58.5%) had major disease-related events (objective disease progression or death). Statistically significant prolongation of PFS can be seen in the TKI+CHM arm (median, 13.5 months; 95% CI, 10.3–16.6 months) compared with the TKI monotherapy (median, 10.9 months; 95% CI, 9.0–12.5 months; HR, 0.68; 95% CI, 0.51–0.90; *P* = 0.0064; **Table 3** **;** **Figure 2A**). A statistically significant improvement in PFS in the TKI+CHM arm versus the TKI arm was observed in patients receiving TKI+CHM as the first-line treatment rather than second-line treatment (**Table 3** **;** **Figure 2B and C**). PFS significantly improved for patients with exon 19 deletions in the TKI+CHM arm (**Table 3** **;** **Figure 2D**), and no obvious difference was observed for patients with exon 21 L858R point mutations between the groups (**Table 3** **;** **Figure 2E**). Analyses of PFS in other patient subgroups, such as females, ≥65 years, icotinib, and never-smokers were consistent with the intent-to-treat (ITT) results (**Table 3**).

**Table 3:** Efficacy outcomes (ITT population).

Outcome	EGFR-TKI+CHM (*N* = 185)	EGFR-TKI+placebo (*N* = 169)	*P* ^a^	HR statistical values^b^	
				HR (95% CI)	*P*
PFS					
Median, months (95% CI)	13.50 (11.20, 16.46)	10.94 (8.97, 12.45)	0.0064	0.68 (0.51, 0.90)	0.0057
Adjusted^c^					
Median PFS by first- or second-line treatment, months (95% CI)					
First line	15.97 (12.09, 16.66)	10.97 (8.94, 13.37)	0.0447	0.78 (0.39, 1.55)	0.4739
Second line	11.43 (9.88, 16.26)	9.23 (6.80, 13.83)	0.0530	1.41 (0.47, 4.21)	0.5391
Median PFS by sex, months (95% CI)					
Male	12.71 (9.86, 29.70)	10.48 (8.7713.47)	0.0523	0.93 (0.51, 1.70)	0.8137
Female	16.07 (11.14, 16.89)	11.24 (8.44, 13.60)	0.0488	1.76 (0.16, 19.57)	0.6463
Median PFS by age, months (95% CI)					
<65 years	12.68 (10.68, 16.07)	10.48 (8.44, 13.37)	0.1158	0.76 (0.55, 1.06)	0.1167
≥65 years	18.83 (11.43,-)	11.56 (8.94, 14.29)	0.0298	0.59 (0.35, 0.98)	0.0436
Median PFS by EGFR-TKI drugs, months (95% CI)					
Gefitinib	16.07 (9.92, 16.66)	10.19 (8.41, 14.29)	0.2284	0.78 (0.29, 2.10)	0.6267
Erlotinib	11.91 (1.94, 31.28)	6.82 (0.72,−)	0.0973	–	–
Icotinib	13.21 (10.97, 18.56)	11.17 (8.90, 13.47)	0.0212	1.27 (0.29, 2.10)	0.5507
Median PFS by EGFR mutation type, months (95% CI)					
Exon 19 deletion	15.97 (11.79, 16.89)	10.87 (8.90, 13.47)	0.0099	0.67 (0.29, 1.57)	0.3593
Exon 21 L858R point mutation	12.68 (9.86, 17.41)	11.24 (8.57, 14.06)	0.1129	1.38 (0.55, 3.48)	0.4940
Other mutation	10.96 (5.52, 16.39)	12.12 (6.01, −)	0.5860	–	–
Median PFS by TCM syndrome, months (95% CI)					
Qi deficiency	12.71 (8.57, 16.43)	10.05 (8.08, 11.70)	0.0557	0.96 (0.40, 2.31)	0.9340
Yin deficiency	13.20 (7.49, 16.46)	12.45 (10.18, 19.81)	0.7819	1.72 (0.40, 7.33)	0.4629
Qi and yin deficiency	16.26 (11.20, 18.83)	10.87 (8.64, 13.60)	0.0281	0.89 (0.32, 2.46)	0.8180
Median PFS by ECOG PS score, months (95% CI)					
0	16.46 (5.22,−)	12.12 (5.52,−)	0.6148	–	–
1−2	13.50 (11.20, 16.43)	10.94 (8.97, 12.45)	0.0081	0.92 (0.51, 1.66)	0.7723
Median PFS by smoking status, months (95% CI)					
Never-smokers	16.07 (11.43, 16.89)	10.97 (8.57, 2.45)	0.0045	0.64 (0.47, 0.88)	0.0053
Present smokers and light former smokers	11.20 (9.17, 5.97)	10.94 (8.97, 6.49)	0.9927	0.96 (0.54, 1.72)	0.9029
Median PFS by stage, months (95% CI)					
III	11.30 (8.18,-)	10.48 (1.08,-)	0.2959	1.14 (0.10, 13.27)	0.9183
IV	13.50 (11.20, 6.46)	10.97 (8.97, 12.94)	0.0087	0.99 (0.54, 1.81)	0.9845
Median TtPD,^d^ months (95% CI)	16.0 (13.57, 18.43)	10.96 (9.45, 12.48)	0.0020	0.64 (0.48, 0.85)	0.0020
OS^e^					
Patients with events No.(%)	42 (22.70)	32 (18.93)	–	–	–
Tumor response, No.(%)^f^					
CR	0 (0.00)	0 (0.00)	–	–	–
PR	119 (64.32)	89 (52.66)	–	–	–
SD	57 (30.81)	74 (43.78)	–	–	–
PD	9 (4.86)	6 (3.56)	–	–	–
ORR(CR+PR)	119 (64.32)	89 (52.66)	0.0260	–	–
DCR(CR+PR+SD)	176 (96.76)	163 (96.45)	1.0000	–	–
Median DoR,^g^ months (95% CI)^h^	9.53 (8.79, 10.23)	8.00 (6.70, 10.29)	0.5000	–	–
Tumor response by first or second line					
First line	79 (63.20)	68 (54.84)	0.1798	–	–
Second line	40 (66.67)	21 (46.67)	0.0398	–	–
Tumor response by EGFR-TKI drugs					
Gefitinib	49 (60.49)	37 (53.62)	0.3965	–	–
Erlotinib	2 (33.33)	0 (0.00)	0.1213	–	–
Icotinib	68 (69.39)	52 (55.32)	0.0441	–	–
Tumor response by EGFR mutation					
Exon 19 deletion	66 (69.47)	51 (51.52)	0.0106	–	–
Exon 21 L858R point mutation	51 (57.95)	36 (53.73)	0.5997	–	–
Other mutation	–	–	–		
Tumor response by TCM syndrome					
Qi deficiency	38 (57.58)	32 (45.71)	0.1666	–	–
Yin deficiency	21 (72.41)	17 (65.38)	0.5773	–	–
Qi and yin deficiency	60 (66.67)	40 (54.79)	0.1217	–	–

EGFR, epidermal growth factor receptor; HR, hazard ratio; ITT, intent to treat; ORR, overall response rate; OS, over survival; PFS, progression-free survival; CR, complete response; PR, partial response; SD, stable disease; PD, progressive disease; DCR, disease control rate; DoR, duration of response.

a A two-sided P-value was derived from log-rank test for PFS and from Fisher’s exact test for tumor response.

b All HRs and corresponding P-values were unadjusted, except as otherwise noted. HR values for TKI+CHM and TKI were derived from a Cox regression analysis, and one-and two-sided P-values were derived from the Wald test from the Cox model.

c Adjusted for EGFR mutation type, age, sex, smoking status, ECOG performance status, stage, TKI drugs, TCM syndrome, and prior chemotherapy therapy.

d Defined as the time from random assignment to the first date of disease progression.

e Defined as the time from random assignment to the date of death from any cause.

f Percentages may not add up to 100% because of rounding.

g Defined as the time from the date of the first CR or PR to the first date of progressive disease (per RECIST version 1.1).

h Analyzed in the ITT population with CR or PR.

The addition of CHM to TKI significantly improved the ORR (64.32% *vs.* 52.66%, *P* = 0.026). For patients taking EGFR-TKI as the first-line therapy, CHM significantly improved the ORR. When used as the second-line treatment, TKI+CHM significantly improved the ORR (66.67% *vs.* 46.67%, *P* = 0.0398). CHM improved the ORR mainly for patients with a 19 Del (69.47% *vs.* 51.52%, *P* = 0.0106) rather than 21L858R. The choice of CHM had no influence on ORR, but icotinib demonstrated a better ORR than the other two TKIs (69.39% *vs.* 55.32%, *P* = 0.0441) (**Table 3**).

### Exon20 T790M

Forty-eight patients (27 cases in the TKI+CHM group and 21 cases in the TKI group) underwent amplification refractory mutation system (ARMS) detection for exon 20 T790M using ctDNA or formalin-fixed paraffin-embedded (FFPE) tissue analysis at the time of tumor progression; 33.33% (9/27) in the TKI+CHM arm and 42.86% (9/21) in the TKI arm were found with exon 20 T790M (*P* = 0.558).

### QoL

**Figure 3:**
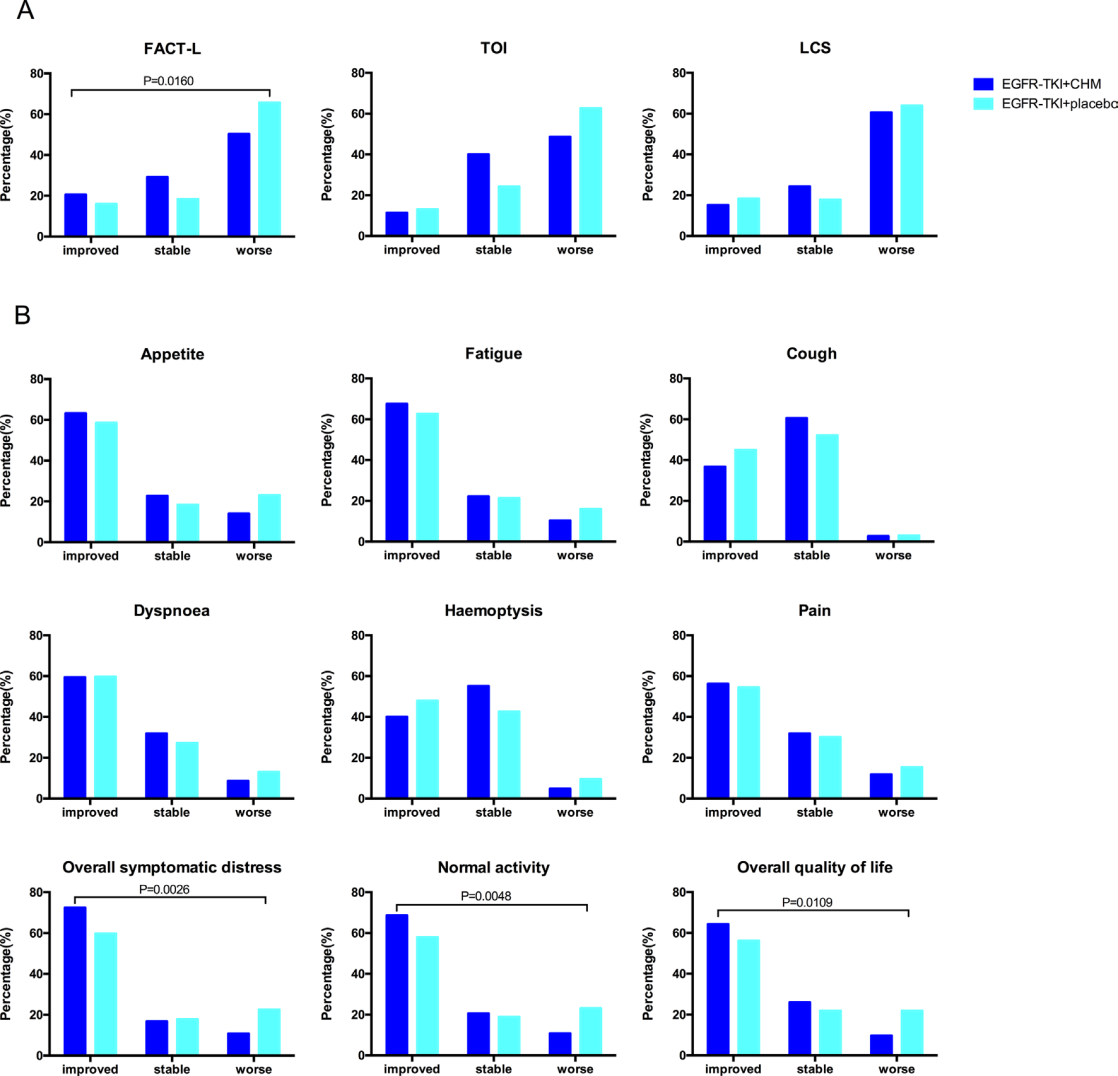
The proportion of patients with different QoL changes during the treatment according to the results of FACT-L, TOI, LCS and the LCSS. The assessments (and improvement rate) were calculated for the FACT-L, TOI, and LCS scores (“improved,” “stabled,” and “worsened”). A clinically relevant improvement was defined as a change from a baseline of six points for FACT-L and TOI and two points for LCS, here maintained for 21 or more days. To demonstrate trends, the baseline score for each item of the LCSS and for the average score was subtracted from the 7-month scores and then categorized as worse (>10 mm), stable (−10 to 10 mm), or improved (<−10 mm). QoL, quality of life; FACT-L, Functional Assessment of Cancer Therapy–Lung; TOI, trial outcome index (the sum of physical, functional well-being, and LCS domains); LCS, lung cancer subscale; LCSS, Lung Cancer Symptom Scale.

There were 326 patients who took part in the questionnaire part of the present study: 92.43% (171/185) in the TKI +CHM arms and 91.72% (155/169) in the TKI arms. The changes in the QoL scores were analyzed for patients at baseline and at 7 months postbaseline QoL assessment (123 in the EGFR-TKI+CHM arm and 116 in the EGFR-TKI+placeboTKI arm). There was no difference in baseline QoL scores in all domains and items between the two groups (*P* > 0.05) (**Supplementary 4** and **5**). The most common symptoms were fatigue for advanced-stage lung cancer patients (mean 37.7 ± 25.1), followed by appetite loss (mean 30.5 ± 24.3), dyspnea (mean 25.7 ± 24.8), and pain (mean 19.2 ± 23.4). The improvement ratios in the FACT-L were significantly higher in the treatment group than in the placebo arm (20.54% [38/185] *vs.* 15.98% [27/169], *P* = 0.0160) **(** **Figure 3A** **)**. For the LCSS pulmonary symptoms score, the patients reported clinically meaningful improvement in their overall QoL, overall symptomatic distress, and normal activity. The improvement ratios in the treatment group were significantly higher than the placebo arm in overall QoL (*P* = 0.0109), overall symptomatic distress (*P* = 0.0026), and normal activity (*P* = 0.0048) **(** **Figure 3B** **)**.

### Safety Outcomes

Mild drug-related TEAEs were reported and included diarrhea, pruritus, skin rash, loss of appetite, and fatigue in the TKI arm **(** **Table 4** **)**. One patient death (9.10%) in the TKI arm caused by AEs related to the study drug (myocardial infarction) occurred within 30 days of the treatment; however, the incidence of study drug-related deaths between arms was similar (*P* = 0.4780). The proportion of patients with study drug-related TEAEs resulting in treatment withdrawal was higher in the TKI arm than in the TKI+CHM arm (*P* = 0.6670) **(** **Table 5** **)**. Here, 83.33% of the patients (10 of 12 patients) undergoing the EGFR-TKI treatment suffered from a treatment interruption as a result of an AE in the TKI+CHM arm and 72.73% (8 of 11 patients) in the TKI-alone arm (*P* = 0.6400). Also, 8.33% of the patients decreased their dose of EGFR-TKI as a result of an AE in the TKI+CHM arm (1 of 12 patients). In the TKI arm, one patient switched to gefitinib instead of icotinib because of the anaphylactic reaction caused by icotinib.

**Table 4:** Most common adverse events of all grades reported by intervention at 7 months of treatment.

Adverse event	EGFR-TKI+CHM (*N* = 115)				EGFR-TKI+placebo (*N* = 99)				*P*-value
	Grade 0	Grade 1	Grade 2	Grade 3 or 4	Grade 0	Grade 1	Grade 2	Grade 3 or 4	
**Fatigue**	94 (81.74%)	20 (17.39%)	1 (0.87%)	0	64 (64.65%)	35 (35.35%)	0 (0.00%)	0	0.0091
**Loss of appetite**	110 (95.65%)	4 (3.48%)	1 (0.87%)	0	86 (86.87%)	12 (12.12%)	1 (1.01%)	0	0.0435
**Diarrhea**	110 (95.65%)	5 (4.35%)	0 (0.00%)	0	86 (86.87%)	13 (13.13%)	0 (0.00%)	0	0.0213
**Pruritus**	97 (84.35%)	17 (14.78%)	1 (0.87)	0	59 (59.60%)	39 (39.39)	1 (1.01%)	0	0.0001
**Skin rash**	93 (80.87%)	19 (16.52%)	3 (2.61%)	0	57 (57.58%)	40 (40.40%)	2 (2.02%)	0	0.0013

**Table 5:** Summary of adverse event rates *n* (%).

	EGFR-TKI+CHM (*N* = 213)	EGFR-TKI+placebo (*N* = 205)	*P*-value
At least one AE* (include SAE**)			
Yes	12 (6.49)§	11 (5.4)※	
No	201 (94.40)	194 (94.60)	1.0000
At least one AE related to the medication (including SAE)			
Yes	9 (75.00)	6 (54.50)	0.4000
No	3 (25.00)	5 (45.50)	
At least one SAE			
Yes	1 (8.30)	2 (18.20)	0.5900
No	11 (91.70)	9 (81.80)	
At least one SAE related to medication			
Yes	1 (8.33)	2 (18.20)	0.5900
No	11 (91.67)	9 (81.80)	
Drop out or stop trial because of AE and SAE			
Yes	3 (25.00)	4 (36.40)	0.6670
No	9 (75.00)	7 (63.60)	
Death because of AE and SAE			
Yes	0 (0.00)	1 (9.10)	0.4780
No	12 (100.00)	10 (90.90)	

*AE, adverse event, **SAE, severe adverse event, §AE: Fever (n = 1), gastrointestinal tract reactions (n = 2), pneumonia (n = 3), increased alanine aminotransferase (ALT) concentration (n = 4), and dizziness (n = 2); ※AE: Gastrointestinal tract reactions (n = 1), herpes zoster (n = 1), pleural effusion (n = 1), heart disease (n = 1), increased ALT concentration (n = 4), anaphylactic reaction (n = 2), and thrombus (n = 1).

## Discussion

To the best of our knowledge, the CATLA is the first prospective head-to-head phase 3 study to examine the efficacy and safety of first- and second-line TKI+CHM versus EGFR-TKI in patients with advanced adenocarcinoma whose tumors harbor EGFR activating mutations. EGFR-TKIs have proved effective in first- or second-line therapy for advanced NSCLC (Mok et al., 2009; Zhou et al., 2011; Shi et al., 2017). A meta-analysis of randomized trials of treatment-native patients reported that EGFR-TKIs statistically significantly prolonged PFS overall, but because of the high rate of crossover at progression, EGFR-TKI had a shorter OS than those who were randomly assigned to chemotherapy (12.8 months, 95% CI : 11.4–14.3 *vs*. 19.8 months, 95% CI: 17.6–21.7) (Lee et al., 2017).

EGFR T790M is a mutation associated with acquired resistance to EGFR-TKI therapy and has been reported in approximately 60% of patients with disease progression after the initial treatment with erlotinib and gefitinib (Yu et al., 2013; Yu et al., 2015). Efforts have been made to delay resistance to EGFR-TKI. Pemetrexed plus gefitinib results in a prolonged PFS (median, 15.8 months; 95% CI, 12.6–18.3 months) when compared with gefitinib alone (median, 10.9 months; 95% CI, 9.7–13.8 months; [HR], 0.68; 95% CI, 0.48–0.96; *P* = 0.014) (Ramalingam et al., 2018). Osimertinib has shown to the potential to prolong PFS to 22.1 months in treatment-naive patients with EGFR advanced NSCLC (Yang et al., 2014). However, these strategies overdraft subsequent chemotherapy and targeted therapy and might not benefit OS.

CHM treatment could be given according to the patients’ physical status, syndrome differentiation, and type of cancer treatment. Patients with lung cancer are receiving the theoretical directions of “treatment of cancer by strengthening antipathogenic ability.” In China, CHM as an adjuvant therapy has shown the potential to reduce chemotherapy toxicity, prolong survival rate, enhance immediate tumor response, and improve Karnofsky performance status (KPS) in advanced NSCLC patients, but its efficacy remains largely unexplored (Li et al., 2013). CHM combined with EGFR-TKI has been demonstrated to be effective in both retrospective and small sample prospective clinical trials (Yang et al., 2014; Hung et al., 2017). The results of the CATLA study conclusively show that TKI+CHM as a first- or second-line therapy provides significantly prolonged PFS and ORR compared with TKI alone in ADC with EGFR activating mutations, here prolonging the median PFS by 5.0 months in the first-line population.

For NSCLC patients with sensitive EGFR mutations, the application of EGFR-TKIs as a first- or second-line therapy did not result in a difference in OS (Mok et al., 2009; Rosell et al., 2012). When used as the first- or second-line therapy, EGFR-TKI resulted in a PFS of 11.0 and 9.2 months and an ORR of 54.8% and 46.7%, which is in accordance with the well-defined efficacy of TKIs (Mok et al., 2009; Mitsudomi et al., 2010; Zhou et al., 2011; Rosell et al., 2012; Shi et al., 2017). To date, no head-to-head trials have been conducted on the difference of efficacy between these EGFR-TKIs (erlotinib, gefitinib, or icotinib) as a first-line therapy in mutated NSCLC populations. By looking at the indirect and integrated comparisons using data from existing clinical trials, there seems to be no difference in efficacy between these TKIs. Twelve phase III RCTs involving 1,821 participants with an EGFR mutation were included in a network meta-analysis, which indicated that erlotinib, gefitinib, and icotinib shared an equivalent efficacy (Liang et al., 2014). In addition, a head-to-head trial between icotinib and gefitinib as a second-line therapy for 68 NSCLC patients with EGFR activating mutations reported that icotinib (*n* = 29) was not inferior to gefitinib (*n* = 39) in terms of PFS (HR 0.78, 95% CI 0.42–1.28; 7.8 months [95% CI 3.7–12.2] and 5.3 months [95% CI 3.7–9.3]; *P* = 0.32, respectively) (Shi et al., 2013). The three kinds of first-generation EGFR-TKIs available in China at the time the study was designed demonstrated comparable efficacy. In the CATLA study, because of the limitations of research funding, the gefitinib, erlotinib, and icotinib treatments applied were paid for by the patients. EGFR-TKI was chosen by the patients; 54.24% (192/354) received icotinib, 42.37% (150/354) chose gefitinib, and only 3.39% (12/354) took erlotinib. Although we did not allocate the type of TKI, we used it as one of the random factors to ensure the groups were balanced. The proportion of patients receiving gefitinib, erlotinib, and icotinib was 40.83%, 3.55%, and 55.62%, respectively, in EGFR-TKI+placebo group and 43.78%, 3.24%, and 52.97%, respectively, in the EGFR+CHM group. There was no statistical difference in TKI selection between the two groups (*P* > 0.05) (**Table 2**). The results show that gefitinib and icotinib have a similar efficacy.

Activating EGFR mutation types included in the current CATLA study trial were exon19 Del (194/354; 54.80%) and exon21 L858R (155/354; 43.79%). Three patients with exon 18 G719X, one with concurrent exon 18 G719X and exon19 Del, and one with concurrent exon19 Del and exon 20 ins were enrolled and included in the ITT group. A significant difference for PFS was observed between the treatments in EGFR-mutated subgroups (exon 19 Del/21 L858R) and TKI+CHM for patients with 19 Del prolonged PFS (median, 15.97 months; 95% CI, 11.79–16.89 months) compared with TKI alone (median, 10.87 months; 95% CI, 8.90–13.47 months; adjusted HR, 0.67; 95% CI, 0.29–1.57; *P* = 0.0099). However, there is no obvious benefit regarding treatment type for 21 L858R (12.68 *vs.* 11.24 months, *P* = 0.1129). In a subgroup analysis, CHM combined with gefitinib seems to prolong the PFS without significance (12.63 months *vs*. 9.67 months, *P* = 0.156), while CHM+icotinib seems to bring no benefits (11.13 months *vs*. 11.87 months, *P* = 0.550, in **Supplementary Tables 6** and **7**). Our study aims to evaluate the clinical efficacy of CHM when combined with TKI, and the mechanism of action of CHM needs further study. Not only did CHM improve the ORR by 20% with TKI in the second-line treatment, but it also improved the ORR by 18%, mainly for patients with 19 Del rather than 21L858R mutation. In general, increasing numbers of studies have shown that patients with different driving mutations have different response to EGFR-TKIs, and the prognosis of exon Del19 is better than that of exon 21L858R, which is consistent with the results of our study (Ke et al., 2017). First, the difference in prognosis between 19 Del and 21 L858R might be related, not only to the efficacy of CHM, but also EGFR-TKIs itself. EGFR has been proved to be more effective for PFS and OS in patients with 19 Del than in those with exon 21L858R mutation (HR_19/21_  =  0.75, 95% CI 0.65 to 0.85; *P* < 0.001) (Zhang et al., 2014). TKI therapy might change the natural history of patients harboring an EGFR mutation, converting Del19 lung cancer from a disease with a poor prognosis to one with a more favorable prognosis (Riely et al., 2006). Second, the difference in intrinsic structural basis between these two kinds of lung cancer explains the possible mechanisms and different drug sensitivity affecting ATP’s binding ability. Del19 removes three to eight residues from the loop leading into the *a*C-helix, whereas the L858R mutation lies in the activation loop of the kinase (Furuyama et al., 2013). Third, the resistance mechanisms (including T790M mutation, mesenchymal-epithelial transition amplification, histological transformation, PIK3CA mutation, and anaplasticlymphoma kinase fusion) differ between Del19 and L858R mutation patients (Ke et al., 2017). The predilection for the T790M mutation in the Del19 population (50.4% *vs.* 36.5%) might help explain why these patients tend to survive longer than patients with the L858R mutation.

The choice of CHM has no influence on the ORR, but icotinib has demonstrated a better ORR than the other two. The ORRs were similar and in accordance with earlier studies. In the current study, it seems that CHM did not change the current situation of the poorer prognosis for patients with exon L858R. Maybe CHM has a greater influence on different drug resistance pathways. However, due to the limitation of this study, the mechanism of CHM delaying TKI resistance need further exploration.

Meanwhile, several studies have shown that CHM could play an important role in EGFR-TKI resistance (Li et al., 2016; Xu et al., 2018). EGFR T790M is a mutation associated with acquired resistance to EGFR-TKI therapy and has been reported in approximately 60% of patients with disease progression after initially being given erlotinib and gefitinib (Yu et al., 2013; Yu et al., 2015). Patients progressed after first-generation EGFR-TKIs were given because of the T790M mutation has a superior efficacy when being treated with osimertinib versus chemotherapy (71% ORR and 10.1 months PFS) (Mok et al., 2017). Thus, a treatment prolonging the PFS of TKI by blocking T790M might not benefit overall OS. We analyzed the EGFR mutation status using ctDNA or tissue at the time of tumor progression to explore the mechanism of CHM in delaying the acquired resistance of TKI. Our results show that CHM delays, rather than blocks, the occurrence of T790M, and patients might still benefit from subsequent osimertinib treatment.

The current study’s drug-related AEs were less frequent in the TKI+CHM arm than in the TKI arm. The most common drug-related TEAEs were diarrhea, pruritus, skin rash, loss of appetite, and fatigue. TCM views health as a balance between yin and yang, and CHM are prescribed accordingly to rebuild that balance. Hence, CHM could help decrease the common adverse reactions because rashes, diarrhea, and so forth may affect patients’ QoL.

Through the current prospective, multicenter, randomized, placebo-controlled randomized clinical study, we confirmed the role of CHM in combination with EGFR-TKI. CHM was also recommended because of its low cost. However, we still need to collect the OS data, which will help determine if CHM can benefit OS.

However, the current study included 91.53% (324/354) stage IV patients; the impact of the imbalance of the clinical staging is relatively small (**Table 1**). Furthermore, there was no difference in the median PFS between stage III and IV (*P* > 0.05) **(** **Table 3** **)**. When we used the stage as a covariate in the multivariate regression analysis, it proved that the stage did not affect the prognosis **(** **Table 3** **)** (*P* > 0.05). In addition, we analyzed the baseline status of brain, bone, and liver metastasis of stage IV patients. The distribution of distant metastasis that could affect PFS and OS was balanced between the two groups.

The current CATLA study provides the first conclusive evidence that the combination of EGFR-TKI and CHM provides a superior ORR and PFS versus EGFR-TKI alone as a first- and second-line treatment in patients whose tumors harbor EGFR activating mutations. However, the current study is limited to relatively small samples and uncontrolled EGFR-TKI types. In addition, clinical staging at the baseline was not well matched. TCM has a long history of clinical observation and application because of its unique theoretical systems. Thus, the active ingredients of the CHM formula and its mechanism in delaying the acquired resistance of EGFR-TKIs remain unknown and need to be explored further.

## Data Availability Statement

The datasets generated for this study are available on request to the corresponding author.

## Ethics Statement

This study was carried out in accordance with the recommendations of NCCN Clinical Practice Guidelines Oncology, Institutional Review Board of LongHua Hospital with written informed consent from all subjects. All subjects gave written informed consent in accordance with the Declaration of Helsinki. The protocol was approved by the Institutional Review Board of LongHua Hospital in Shanghai (IRB no. 2012LCSY018).

## Author Contributions

Conception and design: LJ, JS, ZC, YG, JX, and LX. Financial support: LX; Administrative support: LX; Provision of study materials or patients: LJ, JS, ZC, YG, LB, JY, WZ, AH, GF, YJ, WS, YL, ZZ, PC, JX, and LX; Collection and assembly of data: LJ, JS, ZC, YG, YL, LB, JY, WZ, AH, GF, YJ, WS, YL, ZZ, PC, JX, and LX; Data analysis and interpretation: LJ, YL, PC, JX, and LX; Manuscript writing: All authors; Final approval of manuscript: All authors; Accountable for all aspects of the work: All authors.

## Funding

This study was sponsored by the Shanghai Shenkang Hospital Development Center [no. 16CR1036B], the Shanghai Science and Technology Innovation Project of Traditional Chinese Medicine [no. ZYKC201601020], the Shanghai Municipality Science and Technology Commission Foundation Key Project [no. 16401970700, 11DZ1973200], Young Elite Scientists Sponsorship Program by CAST [CACM-2017-QNRC2-C15], and Special Scientific Research for Traditional Chinese Medicine [no. 201307006].

## Conflict of Interest Statement

The authors declare that the research was conducted in the absence of any commercial or financial relationships that could be construed as a potential conflict of interest.
