# Supplementary material for: Chinese Herbal Medicine Combined With EGFR-TKI in EGFR Mutation-Positive Advanced Pulmonary Adenocarcinoma (CATLA): A Multicenter, Randomized, Double-Blind, Placebo-Controlled Trial
Source: Front Pharmacol. 2019 Jul 2;10:732. doi: 10.3389/fphar.2019.00732 (PMC6614728; doi:10.3389/fphar.2019.00732)
Supplement: Supplementary file 1 [file DataSheet_1.docx]

**Supplementary Table 1: Ingredients of formula I**

| Plant name | Produced from | Dosage (g) | Biological/Pharmacological Activity | Active Components |
| --- | --- | --- | --- | --- |
| Astragalus | Dry rhizoma | 30 | Anti-fatigue, exercise-induced anti-fatigue([Kuo et al., 2009](#_ENREF_23); [Yeh et al., 2014](#_ENREF_58)) | Astragalus flavonoids([Kuo et al., 2009](#_ENREF_23); [Yeh et al., 2014](#_ENREF_58)) Formononetin([Wu et al., 2016](#_ENREF_50)) |
| Codonopsis pilosula | Dry rhizoma | 9 | Immune modulatory([Yongxu and Jicheng, 2008](#_ENREF_59))  Anti-tumor([Fu et al., 2016](#_ENREF_11))  Anti-gut damage([Zhou et al., 2016](#_ENREF_65)) | Polysaccharides, saponins, sesquiterpenes, polyphenolic glycosides, alkaloids, polyacetylenes, essential oils and phytosteroids,([Wang et al., 1995](#_ENREF_48)) codonolactone([Fu et al., 2016](#_ENREF_11)) |
| Atractylodes macrocephala | Dry rhizoma | 12 | Inhibit the ovalbumin (OVA)-mediated allergic diarrhea stimulation of the Th1-type immune responses([Kim et al., 2005](#_ENREF_21))  Reduce diarrhea frequency([Huang et al., 2012](#_ENREF_17))  Anti-inflammatory([Li et al., 2007](#_ENREF_28)) | Atractylenolide, Atractylodes macrophala koidz Polysaccharides([Li et al., 2007](#_ENREF_28)) |
| Poria cocos | Dry sclerotia | 15 | Antioxidant([Tang et al., 2014](#_ENREF_44))  Anti-inflammatory([Jeong et al., 2014](#_ENREF_19)) | Triterpenes([Cheng et al., 2013](#_ENREF_7); [Lee et al., 2017](#_ENREF_27)) |
| Epimedium | Herbal | 15 | Anti-fatigue([Wang et al., 2014](#_ENREF_45))  Antioxidant([Zhao et al., 2014](#_ENREF_64)) | Icariin([Wang et al., 2014](#_ENREF_45))  Phenolic compounds ([Zhao et al., 2014](#_ENREF_64)) |
| Trigonella foenum-graecum | Dry seed | 15 | Antioxidant([Kaviarasan et al., 2007](#_ENREF_20))  Enhanced endurance by the utilization of fatty acids as an energy source([Ikeuchi et al., 2006](#_ENREF_18))  Ameliorates various impairments associated with physical fatigue([Kumar et al., 2013](#_ENREF_22)) | Isoleucine polyphenol, flavonoid, and amino acid contents([Kumar et al., 2013](#_ENREF_22)) |
| Psoralea corylifolia L. | Fruit | 12 | Anti-depressant([Chen et al., 2005](#_ENREF_5))  Anti-tumor([Lv and Liu, 2017](#_ENREF_32)) | Coumarin, flavonoids, terpene phenolic([Xu et al., 2012](#_ENREF_54)) |

**Supplementary Table 2: Ingredients of formula II**

| Plant name | Produced from | Dosage(g) | Biological/Pharmacological Activity | Active Components |
| --- | --- | --- | --- | --- |
| Radix adenophorae | Dry rhizoma | 30 | Suppressed development on inflammation and decreased airway damage by suppressing T cell activity, eosinophilia, and bronchial hyperresponsiveness([Roh et al., 2008](#_ENREF_39))  Enhanced the immune function and protect against exogenous pathogens by activating macrophages([Li et al., 2016](#_ENREF_29)) | Radix Adenophorae Polysaccharide([Li et al., 2016](#_ENREF_29)) |
| Glehnia littoralis | Dry rhizoma | 30 | Promotes neurogenesis([Park et al., 2018](#_ENREF_37))  Anti-tumor([Wu et al., 2018](#_ENREF_49)) | 1-linoloyl-3-palmitoylglycerol, facarindiol, panaxynol, isoimperatorin, β-sitosterol, scopoletin, and umbelliferone([Su et al., 2013](#_ENREF_42)) |
| Radix asparagi | Dry rhizoma | 15 | Anti-inflammatory([Lee et al., 2009](#_ENREF_26))  Stimulation of salivary secretion Xerostomia([Murakami et al., 2009](#_ENREF_36)) | Saponin([Sung et al., 2017](#_ENREF_43)) |
| Ophiopogon japonicus | Dry rhizoma | 15 | Stimulation of salivary secretion([Murakami et al., 2009](#_ENREF_36))  Antioxidant and immunoregulatory([Xiong et al., 2011](#_ENREF_53)) | Homoisoflavonoids ([Chang et al., 2002](#_ENREF_1)) |
| Lilium brownii | Scale leaf | 15 | Enhanced the immune function([Hou et al., 2016](#_ENREF_15))  Anti-tumor([Han and Xie, 2013](#_ENREF_13))  Anti-fatigue([Chun-Lian et al., 2009](#_ENREF_8)) | Lily polysaccharide ([Han and Xie, 2013](#_ENREF_13)) |
| Ligustrum lucidum | Fruit | 12 | Anti-tumor([Hu et al., 2014](#_ENREF_16))  Anti-osteoporosis([Che and Wong, 2015](#_ENREF_2))  Anti-inflammatory([Yoon et al., 2010](#_ENREF_60)) | Polysaccharides([Yoon et al., 2010](#_ENREF_60))  Ursolic acid([Xia et al., 2012](#_ENREF_51)) |

**Supplementary Table 3: Ingredients of formula III**

| Plant name | Produced from | Dosage (g) | Biological /Pharmacological Activity | Active Components |
| --- | --- | --- | --- | --- |
| Prunella vulgaris L. | Dry spikes | 7.5 | Anti-tumor([Han et al., 2018](#_ENREF_14))  Anti-inflammatory([Rocha et al., 2015](#_ENREF_38))  Stimulates macrophage activation ([Han et al., 2009](#_ENREF_12))  Enhances apoptosis([Moon et al., 2010](#_ENREF_35))  Against aging([Zhang et al., 2018](#_ENREF_62)) | Rosmarinic, ellagic, and caffeic acids([Lamaison et al., 1991](#_ENREF_25))  Phenolic acids, flavonoids, coumarins, triterpenes, volatile oil, polysaccharides([Feng et al., 2010](#_ENREF_9); [Moon et al., 2010](#_ENREF_35)) |
| Arisaema heterophyllum Blume | Dry rhizoma | 15 | Anti-tumor, apoptosis, and autophagy ([Feng et al., 2016](#_ENREF_10)) | Glycerol monostearic acid, ß-sitosterol, daucosterol, and succinic acid([Yang et al., 2003](#_ENREF_55)) |
| Rhizoma amorphophalli | Dry tubers | 15 | Anti-tumor([Sawai et al., 2018](#_ENREF_40)) ([Mingxiang, 2014](#_ENREF_34)) | Konjac glucomannan([Mingxiang, 2014](#_ENREF_34)) |
| Cremastra appendiculata | Dry pseudobulb | 7.5 | Anti-tumor([Liu et al., 2016](#_ENREF_31)) | Biphenanthrenes([Liu et al., 2016](#_ENREF_31)) |
| Euphorbia helioscopia | Herbal | 7.5 | Anti-tumor([Cheng et al., 2015](#_ENREF_6)) | Jatrophane-type diterpenoids([Shang et al., 2011](#_ENREF_41))  Diterpenoids([Mai et al., 2018](#_ENREF_33)) |
| Selaginella doederleinii Hieron | Herbal | 15 | Anti-tumor([Yao et al., 2017](#_ENREF_56))  Anti-inflammatory, antioxidant, anti-fungal, and anti-virus activity([Li et al., 2014](#_ENREF_30)) | Biflavonoids([Li et al., 2014](#_ENREF_30)) |
| Salvia chinensis Benth | Herbal | 15 | Anti-tumor([Wang et al., 2017](#_ENREF_47)) | Total flavonoids([Xiang et al., 2013](#_ENREF_52))  Protocatechualdehyde([Wang et al., 2017](#_ENREF_47)) |
| Paris polyphylla | Dry rhizoma | 7.5 | Anti-tumor([Wang et al., 2016](#_ENREF_46)) | Polyphyllin VII([Zhang et al., 2016](#_ENREF_61)) |
| Jujube date | Fruit | 4.5 | Stimulated erythropoietin([Chen et al., 2014b](#_ENREF_4); [Lam et al., 2016](#_ENREF_24))  Antioxidative, anti-tumor([Ye and Son, 2012](#_ENREF_57))  Anti-fatigue([Zhang and Mao-Dong, 2006](#_ENREF_63))  Anti-inflammatory([Chen et al., 2014a](#_ENREF_3)) | Polysaccharides([Zhang and Mao-Dong, 2006](#_ENREF_63)) |

Reference

Chang, J.M., Shen, C.C., Huang, Y.L., Chien, M.Y., Ou, J.C., Shieh, B.J., et al. (2002). Five new homoisoflavonoids from the tuber of Ophiopogon japonicus. *J Nat Prod* 65(11)**,** 1731-1733.

Che, C.T., and Wong, M.S. (2015). Ligustrum lucidum and its Constituents: A Mini-Review on the Anti-Osteoporosis Potential. *Nat Prod Commun* 10(12)**,** 2189-2194.

Chen, J., Du, C.Y., Lam, K.Y., Zhang, W.L., Lam, C.T., Yan, A.L., et al. (2014a). The standardized extract of Ziziphus jujuba fruit (jujube) regulates pro-inflammatory cytokine expression in cultured murine macrophages: suppression of lipopolysaccharide-stimulated NF-kappaB activity. *Phytother Res* 28(10)**,** 1527-1532. doi: 10.1002/ptr.5160.

Chen, J., Lam, C.T., Kong, A.Y., Zhang, W.L., Zhan, J.Y., Bi, C.W., et al. (2014b). The extract of Ziziphus jujuba fruit (jujube) induces expression of erythropoietin via hypoxia-inducible factor-1alpha in cultured Hep3B cells. *Planta Med* 80(17)**,** 1622-1627. doi: 10.1055/s-0034-1383049.

Chen, Y., Kong, L.D., Xia, X., Kung, H.F., and Zhang, L. (2005). Behavioral and biochemical studies of total furocoumarins from seeds of Psoralea corylifolia in the forced swimming test in mice. *J Ethnopharmacol* 96(3)**,** 451-459. doi: 10.1016/j.jep.2004.09.033.

Cheng, J., Han, W., Wang, Z., Shao, Y., Wang, Y., Zhang, Y., et al. (2015). Hepatocellular Carcinoma Growth Is Inhibited by Euphorbia helioscopia L. Extract in Nude Mice Xenografts. *Biomed Res Int* 2015**,** 601015. doi: 10.1155/2015/601015.

Cheng, S., Eliaz, I., Lin, J., Thyagarajan-Sahu, A., and Sliva, D. (2013). Triterpenes from Poria cocos suppress growth and invasiveness of pancreatic cancer cells through the downregulation of MMP-7. *Int J Oncol* 42(6)**,** 1869-1874. doi: 10.3892/ijo.2013.1902.

Chun-Lian, H.E., Yang, X.H., Huang, H., and Yang, Q.Z. (2009). Anti-weary Pharmacological Action of Lily Polysaccharide. *Journal of Hunan Normal University*.

Feng, L., Jia, X.B., Shi, F., and Chen, Y. (2010). Identification of two polysaccharides from Prunella vulgaris L. and evaluation on their anti-lung adenocarcinoma activity. *Molecules* 15(8)**,** 5093-5103. doi: 10.3390/molecules15085093.

Feng, L.X., Sun, P., Mi, T., Liu, M., Liu, W., Yao, S., et al. (2016). Agglutinin isolated from Arisema heterophyllum Blume induces apoptosis and autophagy in A549 cells through inhibiting PI3K/Akt pathway and inducing ER stress. *Chin J Nat Med* 14(11)**,** 856-864. doi: 10.1016/S1875-5364(16)30102-9.

Fu, J., Ke, X., Tan, S., Liu, T., Wang, S., Ma, J., et al. (2016). The natural compound codonolactone attenuates TGF-beta1-mediated epithelial-to-mesenchymal transition and motility of breast cancer cells. *Oncol Rep* 35(1)**,** 117-126. doi: 10.3892/or.2015.4394.

Han, E.H., Choi, J.H., Hwang, Y.P., Park, H.J., Choi, C.Y., Chung, Y.C., et al. (2009). Immunostimulatory activity of aqueous extract isolated from Prunella vulgaris. *Food Chem Toxicol* 47(1)**,** 62-69. doi: 10.1016/j.fct.2008.10.010.

Han, H.P., and Xie, H.C. (2013). A study on the extraction and purification process of lily polysaccharide and its anti-tumor effect. *Afr J Tradit Complement Altern Med* 10(6)**,** 485-489.

Han, Y.H., Kee, J.Y., and Hong, S.H. (2018). Rosmarinic Acid Activates AMPK to Inhibit Metastasis of Colorectal Cancer. *Front Pharmacol* 9**,** 68. doi: 10.3389/fphar.2018.00068.

Hou, R., Chen, J., Yue, C., Li, X., Liu, J., Gao, Z., et al. (2016). Modification of lily polysaccharide by selenylation and the immune-enhancing activity. *Carbohydr Polym* 142**,** 73-81. doi: 10.1016/j.carbpol.2016.01.032.

Hu, B., Du, Q., Deng, S., An, H.M., Pan, C.F., Shen, K.P., et al. (2014). Ligustrum lucidum Ait. fruit extract induces apoptosis and cell senescence in human hepatocellular carcinoma cells through upregulation of p21. *Oncol Rep* 32(3)**,** 1037-1042. doi: 10.3892/or.2014.3312.

Huang, C.W., Lee, T.T., Shih, Y.C., and Yu, B. (2012). Effects of dietary supplementation of Chinese medicinal herbs on polymorphonuclear neutrophil immune activity and small intestinal morphology in weanling pigs. *J Anim Physiol Anim Nutr (Berl)* 96(2)**,** 285-294. doi: 10.1111/j.1439-0396.2011.01151.x.

Ikeuchi, M., Yamaguchi, K., Koyama, T., Sono, Y., and Yazawa, K. (2006). Effects of fenugreek seeds (Trigonella foenum greaecum) extract on endurance capacity in mice. *J Nutr Sci Vitaminol (Tokyo)* 52(4)**,** 287-292.

Jeong, J.W., Lee, H.H., Han, M.H., Kim, G.Y., Hong, S.H., Park, C., et al. (2014). Ethanol extract of Poria cocos reduces the production of inflammatory mediators by suppressing the NF-kappaB signaling pathway in lipopolysaccharide-stimulated RAW 264.7 macrophages. *BMC Complement Altern Med* 14**,** 101. doi: 10.1186/1472-6882-14-101.

Kaviarasan, S., Naik, G.H., Gangabhagirathi, R., Anuradha, C.V., and Priyadarsini, K.I. (2007). In vitro studies on antiradical and antioxidant activities of fenugreek ( Trigonella foenum graecum ) seeds. *Food Chemistry* 103(1)**,** 31-37.

Kim, S.H., Jung, H.N., Lee, K.Y., Kim, J., Lee, J.C., and Jang, Y.S. (2005). Suppression of Th2-type immune response-mediated allergic diarrhea following oral administration of traditional Korean medicine: Atractylodes macrocephala Koidz. *Immunopharmacol Immunotoxicol* 27(2)**,** 331-343. doi: 10.1081/IPH-200067950.

Kumar, G.P., Anand, T., Singsit, D., Khanum, F., and Anilakumar, K.R. (2013). Evaluation of antioxidant and anti-fatigue properties of Trigonella foenum-graecum L. in rats subjected to weight loaded forced swim test. *Pharmacognosy Journal* 5(2)**,** 66–71.

Kuo, Y.H., Tsai, W.J., Loke, S.H., Wu, T.S., and Chiou, W.F. (2009). Astragalus membranaceus flavonoids (AMF) ameliorate chronic fatigue syndrome induced by food intake restriction plus forced swimming. *J Ethnopharmacol* 122(1)**,** 28-34. doi: 10.1016/j.jep.2008.11.025.

Lam, C.T.W., Chan, P.H., Lee, P.S.C., Lau, K.M., Kong, A.Y.Y., Gong, A.G.W., et al. (2016). Chemical and biological assessment of Jujube (Ziziphus jujuba)-containing herbal decoctions: Induction of erythropoietin expression in cultures. *J Chromatogr B Analyt Technol Biomed Life Sci* 1026**,** 254-262. doi: 10.1016/j.jchromb.2015.09.021.

Lamaison, J.L., Petitjean-Freytet, C., and Carnat, A. (1991). Medicinal Lamiaceae with antioxidant properties, a potential source of rosmarinic acid. *Pharm Acta Helv* 66(7)**,** 185-188.

Lee, D.Y., Choo, B.K., Yoon, T., Cheon, M.S., Lee, H.W., Lee, A.Y., et al. (2009). Anti-inflammatory effects of Asparagus cochinchinensis extract in acute and chronic cutaneous inflammation. *J Ethnopharmacol* 121(1)**,** 28-34. doi: 10.1016/j.jep.2008.07.006.

Lee, S.R., Lee, S., Moon, E., Park, H.J., Park, H.B., and Kim, K.H. (2017). Bioactivity-guided isolation of anti-inflammatory triterpenoids from the sclerotia of Poria cocos using LPS-stimulated Raw264.7 cells. *Bioorg Chem* 70**,** 94-99. doi: 10.1016/j.bioorg.2016.11.012.

Li, C.Q., He, L.C., Dong, H.Y., and Jin, J.Q. (2007). Screening for the anti-inflammatory activity of fractions and compounds from Atractylodes macrocephala koidz. *J Ethnopharmacol* 114(2)**,** 212-217. doi: 10.1016/j.jep.2007.08.002.

Li, J.W., Liu, Y., Li, B.H., Wang, Y.Y., Wang, H., and Zhou, C.L. (2016). A polysaccharide purified from Radix Adenophorae promotes cell activation and pro-inflammatory cytokine production in murine RAW264.7 macrophages. *Chin J Nat Med* 14(5)**,** 370-376. doi: 10.3724/sp.j.1009.2016.00370.

Li, S., Zhao, M., Li, Y., Sui, Y., Yao, H., Huang, L., et al. (2014). Preparative isolation of six anti-tumour biflavonoids from Selaginella doederleinii Hieron by high-speed counter-current chromatography. *Phytochem Anal* 25(2)**,** 127-133. doi: 10.1002/pca.2478.

Liu, L., Li, J., Zeng, K.W., Jiang, Y., and Tu, P.F. (2016). Five New Biphenanthrenes from Cremastra appendiculata. *Molecules* 21(8). doi: 10.3390/molecules21081089.

Lv, L., and Liu, B. (2017). Antitumor effects of bakuchiol on human gastric carcinoma cell lines are mediated through PI3K/AKT and MAPK signaling pathways. *Mol Med Rep* 16(6)**,** 8977-8982. doi: 10.3892/mmr.2017.7696.

Mai, Z.P., Ni, G., Liu, Y.F., Li, Y.H., Li, L., Li, J.Y., et al. (2018). Secoheliosphanes A and B and Secoheliospholane A, Three Diterpenoids with Unusual seco-Jatrophane and seco-Jatropholane Skeletons from Euphorbia helioscopia. *The Journal of organic chemistry* 83(1)**,** 167-173. doi: 10.1021/acs.joc.7b02558.

Mingxiang, H.E. (2014). Study on Antitumor Activity of Konjac Glucomannan from Rhizoma Amorphophalli. *Journal of Food Science & Technology*.

Moon, D.O., Kim, M.O., Lee, J.D., Choi, Y.H., and Kim, G.Y. (2010). Rosmarinic acid sensitizes cell death through suppression of TNF-alpha-induced NF-kappaB activation and ROS generation in human leukemia U937 cells. *Cancer Lett* 288(2)**,** 183-191. doi: 10.1016/j.canlet.2009.06.033.

Murakami, M., Wei, M.X., Ding, W., and Zhang, Q.D. (2009). Effects of Chinese herbs on salivary fluid secretion by isolated and perfused rat submandibular glands. *World J Gastroenterol* 15(31)**,** 3908-3915.

Park, J.H., Shin, B.N., Ahn, J.H., Cho, J.H., Lee, T.K., Lee, J.C., et al. (2018). Glehnia littoralis Extract Promotes Neurogenesis in the Hippocampal Dentate Gyrus of the Adult Mouse through Increasing Expressions of Brain-Derived Neurotrophic Factor and Tropomyosin-Related Kinase B. *Chin Med J (Engl)* 131(6)**,** 689-695. doi: 10.4103/0366-6999.226894.

Rocha, J., Eduardo-Figueira, M., Barateiro, A., Fernandes, A., Brites, D., Bronze, R., et al. (2015). Anti-inflammatory effect of rosmarinic acid and an extract of Rosmarinus officinalis in rat models of local and systemic inflammation. *Basic Clin Pharmacol Toxicol* 116(5)**,** 398-413. doi: 10.1111/bcpt.12335.

Roh, S.S., Kim, S.H., Lee, Y.C., and Seo, Y.B. (2008). Effects of radix adenophorae and cyclosporine A on an OVA-induced murine model of asthma by suppressing to T cells activity, eosinophilia, and bronchial hyperresponsiveness. *Mediators Inflamm* 2008**,** 781425. doi: 10.1155/2008/781425.

Sawai, S., Mokhtar, M.S., Safwani, W., and Ramasamy, T.S. (2018). Suppression of the Viability and Proliferation of HepG2 Hepatocellular Carcinoma Cell Line by Konjac Glucomannan. *Anticancer Agents Med Chem*. doi: 10.2174/1871520618666180307143229.

Shang, S., Shu-Zhen, M.U., Huang, Y.M., Jin, H.L., and Hao, X.J. (2011). Extraction and Identification of Jatrophane-type Diterpenoids from Euphorbia helioscopia L. *Journal of Mountain Agriculture & Biology*.

Su, X., Li, X., Tao, H., Zhou, J., Wu, T., Chou, G., et al. (2013). Simultaneous isolation of seven compounds from Glehnia littoralis roots by off-line overpressured layer chromatography guided by a TLC antioxidant autographic assay. *J Sep Sci* 36(21-22)**,** 3644-3650. doi: 10.1002/jssc.201300523.

Sung, J.E., Lee, H.A., Kim, J.E., Yun, W.B., An, B.S., Yang, S.Y., et al. (2017). Saponin-enriched extract of Asparagus cochinchinensis alleviates airway inflammation and remodeling in ovalbumin-induced asthma model. *Int J Mol Med* 40(5)**,** 1365-1376. doi: 10.3892/ijmm.2017.3147.

Tang, J., Nie, J., Li, D., Zhu, W., Zhang, S., Ma, F., et al. (2014). Characterization and antioxidant activities of degraded polysaccharides from Poria cocos sclerotium. *Carbohydr Polym* 105**,** 121-126. doi: 10.1016/j.carbpol.2014.01.049.

Wang, B., Yan, F., and Cai, L. (2014). Anti-fatigue properties of icariin from Epimedium brevicornum. *Biomedical Research* 25(3)**,** 297-302.

Wang, C.W., Tai, C.J., Choong, C.Y., Lin, Y.C., Lee, B.H., Shi, Y.C., et al. (2016). Aqueous Extract of Paris polyphylla (AEPP) Inhibits Ovarian Cancer via Suppression of Peroxisome Proliferator-Activated Receptor-Gamma Coactivator (PGC)-1alpha. *Molecules* 21(6). doi: 10.3390/molecules21060727.

Wang, N., Tan, H.Y., Chan, Y.T., Guo, W., Li, S., and Feng, Y. (2017). Identification of WT1 as determinant of heptatocellular carcinoma and its inhibition by Chinese herbal medicine Salvia chinensis Benth and its active ingredient protocatechualdehyde. *Oncotarget* 8(62)**,** 105848-105859. doi: 10.18632/oncotarget.22406.

Wang, Z.T., Ma, G.Y., Tu, P.F., Xu, G.J., and Ng, T.B. (1995). Chemotaxonomic study of Codonopsis (family Campanulaceae) and its related genera. *Biochemical Systematics & Ecology* 23(7-8)**,** 809-812.

Wu, J., Gao, W., Song, Z., Xiong, Q., Xu, Y., Han, Y., et al. (2018). Anticancer activity of polysaccharide from Glehnia littoralis on human lung cancer cell line A549. *Int J Biol Macromol* 106**,** 464-472. doi: 10.1016/j.ijbiomac.2017.08.033.

Wu, J., Ke, X., Ma, N., Wang, W., Fu, W., Zhang, H., et al. (2016). Formononetin, an active compound of Astragalus membranaceus (Fisch) Bunge, inhibits hypoxia-induced retinal neovascularization via the HIF-1alpha/VEGF signaling pathway. *Drug Des Devel Ther* 10**,** 3071-3081. doi: 10.2147/dddt.s114022.

Xia, E.Q., Yu, Y.Y., Xu, X.R., Deng, G.F., Guo, Y.J., and Li, H.B. (2012). Ultrasound-assisted extraction of oleanolic acid and ursolic acid from Ligustrum lucidum Ait. *Ultrason Sonochem* 19(4)**,** 772-776. doi: 10.1016/j.ultsonch.2011.11.014.

Xiang, M., Su, H., Hu, Y., Hu, Y., Yang, T., and Shu, G. (2013). Chemical composition of total flavonoids from Salvia chinensia Benth and their pro-apoptotic effect on hepatocellular carcinoma cells: potential roles of suppressing cellular NF-kappaB signaling. *Food Chem Toxicol* 62**,** 420-426. doi: 10.1016/j.fct.2013.09.008.

Xiong, S.L., Li, A., Huang, N., Lu, F., and Hou, D. (2011). Antioxidant and immunoregulatory activity of different polysaccharide fractions from tuber of Ophiopogon japonicus. *Carbohydrate Polymers* 86(3)**,** 1273-1280.

Xu, M.J., Wu, B., Ding, T., Chu, J.H., Li, C.Y., Zhang, J., et al. (2012). Simultaneous characterization of prenylated flavonoids and isoflavonoids in Psoralea corylifolia L. by liquid chromatography with diode-array detection and quadrupole time-of-flight mass spectrometry. *Rapid Commun Mass Spectrom* 26(19)**,** 2343-2358. doi: 10.1002/rcm.6361.

Yang, Z.L., Wei, Y.J., and Wen cai, Y.E. (2003). Studies on the chemical constituents of Arisaema heterophyllum blume. *Chinese Traditional Patent Medicine* 25(3)**,** 228-229.

Yao, H., Chen, B., Zhang, Y., Ou, H., Li, Y., Li, S., et al. (2017). Analysis of the Total Biflavonoids Extract from Selaginella doederleinii by HPLC-QTOF-MS and Its In Vitro and In Vivo Anticancer Effects. *Molecules* 22(2). doi: 10.3390/molecules22020325.

Ye, J.K., and Son, D.Y. (2012). Hot water leaves extracts of Zizyphus jujube exert antioxidative effects in vitro and cytotoxicity in human cancer cell lines. *Horticulture Environment & Biotechnology* 52(6)**,** 635-640.

Yeh, T.S., Chuang, H.L., Huang, W.C., Chen, Y.M., Huang, C.C., and Hsu, M.C. (2014). Astragalus membranaceus improves exercise performance and ameliorates exercise-induced fatigue in trained mice. *Molecules* 19(3)**,** 2793-2807. doi: 10.3390/molecules19032793.

Yongxu, S., and Jicheng, L. (2008). Structural characterization of a water-soluble polysaccharide from the roots of Codonopsis pilosula and its immunity activity. *Int J Biol Macromol* 43(3)**,** 279-282. doi: 10.1016/j.ijbiomac.2008.06.009.

Yoon, T., Lee, D.Y., Lee, A.Y., Choi, G., Choo, B.K., and Kim, H.K. (2010). Anti-inflammatory effects of Glehnia littoralis extract in acute and chronic cutaneous inflammation. *Immunopharmacol Immunotoxicol* 32(4)**,** 663-670. doi: 10.3109/08923971003671108.

Zhang, C., Jia, X., Bao, J., Chen, S., Wang, K., Zhang, Y., et al. (2016). Polyphyllin VII induces apoptosis in HepG2 cells through ROS-mediated mitochondrial dysfunction and MAPK pathways. *BMC Complement Altern Med* 16**,** 58. doi: 10.1186/s12906-016-1036-x.

Zhang, M., Hwang, E., Lin, P., Gao, W., Ngo, H.T.T., and Yi, T.H. (2018). Prunella vulgaris L. exerts a protective effect against extrinsic aging via NF-kappaB, MAPKs, AP-1, and TGF-beta/Smad signaling pathways in UVB-aged normal human dermal fibroblasts. *Rejuvenation Res*. doi: 10.1089/rej.2017.1971.

Zhang, Z., and Mao-Dong, W.U. (2006). Protective effect of jujube dates polysaccharide on chemical liver injury in mice and antifatigue effect. *Journal of Nanjing Agricultural University* 29(1)**,** 94-97.

Zhao, Y., Hou, Y., Tang, G., Cai, E., Liu, S., Yang, H., et al. (2014). Optimization of Ultrasonic Extraction of Phenolic Compounds from Epimedium brevicornum Maxim Using Response Surface Methodology and Evaluation of Its Antioxidant Activities In Vitro. *J Anal Methods Chem* 2014**,** 864654. doi: 10.1155/2014/864654.

Zhou, Y., Liu, Z., Chen, Y., and Jin, L.H. (2016). Identification of the protective effects of traditional medicinal plants against SDS-induced Drosophila gut damage. *Exp Ther Med* 12(4)**,** 2671-2680. doi: 10.3892/etm.2016.3641.
