# Supplementary material for: Chinese Herbal Medicine Combined With EGFR-TKI in EGFR Mutation-Positive Advanced Pulmonary Adenocarcinoma (CATLA): A Multicenter, Randomized, Double-Blind, Placebo-Controlled Trial
Source: Front Pharmacol. 2019 Jul 2;10:732. doi: 10.3389/fphar.2019.00732 (PMC6614728; doi:10.3389/fphar.2019.00732)
Supplement: Supplementary file 2 [file DataSheet_2.docx]

**Supplementary Table 4 FACT-L score at baseline**

| Items | EGFR-TKI+CHM |  | EGFR-TKI+placebo | *P*-value* |
| --- | --- | --- | --- | --- |
|  | N=171(14 missing)  Mean±SD |  | N=155(14 missing)  Mean±SD |  |
| Physical well-being  (PWB) | 6.9±4.7 |  | 6.6±4.9 | 0.3186 |
| Social/family well-being (SWB) | 22.3±6.3 |  | 22.3±7.1 | 0.4383 |
| Emotional well-being  (EWB) | 7.3±4.6 |  | 7.3±4.1 | 0.6798 |
| Functional well-being, (FWB) | 13.4±6.3 |  | 14.3±6.3 | 0.1354 |
| Lung Cancer Subscale (LCS) | 13.5±3.9 |  | 13.4±4.3 | 0.6785 |
| FACT-L total score  (FACT-L) | 63.4±13.9 |  | 63.9±15.3 | 0.2746 |
| Trial Outcome Index  (TOI) | 33.7±8.1 |  | 34.3±9.0 | 0.2955 |

*: Wilcoxon test
