# Supplementary material for: Chinese Herbal Medicine Combined With EGFR-TKI in EGFR Mutation-Positive Advanced Pulmonary Adenocarcinoma (CATLA): A Multicenter, Randomized, Double-Blind, Placebo-Controlled Trial
Source: Front Pharmacol. 2019 Jul 2;10:732. doi: 10.3389/fphar.2019.00732 (PMC6614728; doi:10.3389/fphar.2019.00732)
Supplement: Supplementary file 3 [file DataSheet_3.doc]

**Supplementary Table 5. LCSS score at baseline**

| Items | EGFR-TKI+CHM |  | EGFR-TKI+placebo | *P*-value* |
| --- | --- | --- | --- | --- |
|  | N=171(14 missing)  Mean±SD |  | N=155(14missing)  Mean±SD |
| Appetite | 30.5±24.3 |  | 29.0±26.0 | 0.3546 |
| Fatigue | 37.7±25.1 |  | 37.0±23.0 | 0.6982 |
| Cough | 1.8±3.8 |  | 2.1±5.4 | 0.8607 |
| Dyspnea | 25.7±24.8 |  | 27.5±23.8 | 0.3036 |
| Hemoptysis | 4.9±15.2 |  | 3.5±11.5 | 0.2433 |
| Pain | 19.2±23.4 |  | 20.8±25.7 | 0.7565 |
| Overall symptomatic Distress | 46.2±26.1 |  | 43.0±26.2 | 0.2524 |
| Normal activity | 44.7±26.1 |  | 43.7±26.2 | 0.5651 |
| Overall quality of life | 41.9±23.3 |  | 40.8±24.8 | 0.4743 |

*: Wilcoxon test
