# Supplementary material for: Chinese Herbal Medicine Combined With EGFR-TKI in EGFR Mutation-Positive Advanced Pulmonary Adenocarcinoma (CATLA): A Multicenter, Randomized, Double-Blind, Placebo-Controlled Trial
Source: Front Pharmacol. 2019 Jul 2;10:732. doi: 10.3389/fphar.2019.00732 (PMC6614728; doi:10.3389/fphar.2019.00732)
Supplement: Supplementary file 4 [file DataSheet_4.docx]

**Supplementary Table 6. Patient demographic and baseline disease characteristics (Exon21 L858R subgroup population)**

| EGFR-TKI drugs | EGFR-TKI+ CHM  (N=88) | EGFR-TKI+placebo  (N=67) | Total  (N=155) |
| --- | --- | --- | --- |
| Gefitinib | 42(47.7%) | 26(38.8%) | 68(43.9%) |
| Erlotinib | 2(2.3%) | 3(4.5%) | 5(3.2%) |
| Icotinib | 44(50.0%) | 38(56.7%) | 82(52.9%) |

**Supplementary Table 7. The efficacy of the different TKIs in the treatment of Exon21 L858R**

| Outcome | EGFR-TKI+CHM  (n=88) | EGFR-TKI+placebo  (n=67) | *P* ^a^ | HR Statistical Values^b^ | |
| --- | --- | --- | --- | --- | --- |
|  |  |  |  | HR (95% CI) | *P* |
| Median PFS by EGFR-TKI drugs, months (95% CI) | | | | | |
| Gefitinib | 12.63(5.45,19.82) | 9.67(6.48,12.85) | 0.156 | 0.632(0.333,1.198) | 0.1006 |
| Icotinib | 11.13(8.13,14.13) | 12.87(10.12,15.62) | 0.550 | 0.833(0.456,1.520) | 0.5051 |

Note: ^a^ A two-sided P-value was derived from log-rank test for PFS.

^b^ All HRs and corresponding *P*-values were unadjusted, except as otherwise noted. HR values for TKI+CHM and TKI were derived from a Cox regression analysis, and one-and two-sided P-values were derived from the Wald test from the Cox model.
