# Supplementary figures and images for: Chinese Herbal Medicine Combined With EGFR-TKI in EGFR Mutation-Positive Advanced Pulmonary Adenocarcinoma (CATLA): A Multicenter, Randomized, Double-Blind, Placebo-Controlled Trial
Source: Front Pharmacol. 2019 Jul 2;10:732. doi: 10.3389/fphar.2019.00732 (PMC6614728; doi:10.3389/fphar.2019.00732)

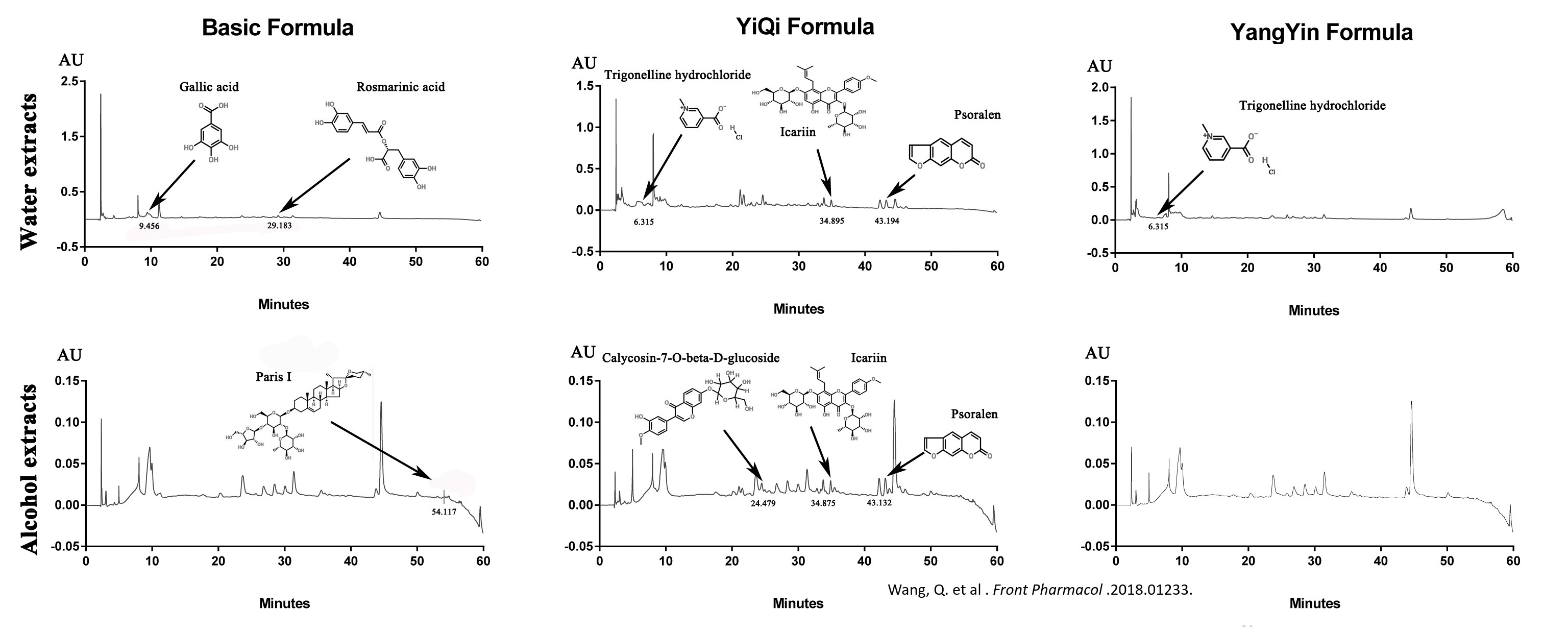

Supplement: Supplementary file 5 [file Image_1.tif]
